# Placebo effects on the quantity and quality of relaxation training

**DOI:** 10.1177/1359105320954238

**Published:** 2020-09-01

**Authors:** Carina Höfler, Florian Osmani, Anne Schienle

**Affiliations:** Department of Clinical Psychology, University of Graz, Graz, Austria

**Keywords:** app-assisted approach, compliance, placebo, relaxation training

## Abstract

Many people find it difficult to practice progressive muscle relaxation (PMR) regularly. We attempted to improve relaxation quantity (i.e. adherence), and relaxation quality via placebo. A total of 100 women were randomly assigned to a standard group, which practiced PMR at home every day for two weeks, or a placebo group, which practiced PMR for two weeks with additional daily placebo treatment. To monitor adherence to relaxation practice, we used a smartphone app. The placebo group practiced more often than the standard group. Both groups did not differ in their reported relaxation level after the daily exercises.

## Introduction

Relaxation training has numerous areas of application. Patients with mental health problems (e.g. symptoms of anxiety disorders, depression) and patients with physical health problems (e.g. high blood pressure, pain) can benefit from practicing relaxation techniques (for results of meta-analyses see Manzoni et al., 2008; Stetter and Kupper, 2002). Relaxation training can also be useful for healthy people to reduce feelings of stress, and to improve somatic and mental well-being (Stetter and Kupper, 2002).

All relaxation techniques elicit a generic ‘relaxation response’ that includes the reduction of muscle tension. This somatic relaxation response is accompanied by positive emotional changes (e.g. feelings of calmness; reduction of arousal). All relaxation techniques are based on the systematic practice of psychomotor relaxation routines (Petermann and Vaitl, 2014). Only with systematic training, stable relaxation effects can be achieved. However, many people find it difficult to practice regularly and as a consequence do not experience (sufficient) relaxation (e.g. Taylor et al., 1983).

Therefore, the present study investigated an intervention to improve the adherence to practice a relaxation technique. The chosen approach was placebo treatment. A placebo is defined as ‘a substance or procedure . . . that is objectively without specific activity for the condition being treated’ (Moerman and Jonas, 2002, p. 471). The most commonly studied placebo phenomenon is ‘placebo analgesia’, during which a patient receives a pharmacologically ineffective intervention (e.g. a pill filled with sugar) with the verbal suggestion that this is a pain-reducing treatment. Several studies have demonstrated that this approach leads to pain relief as well as to altered activation in pain-sensitive brain regions (e.g. Wager and Atlas, 2015).

Placebos have also been used in other areas, for example, to reduce the intensity of negative feelings such as anxiety and disgust (e.g. Petrovic et al., 2005; Schienle et al., 2014), to improve physical performance (e.g. Beedie and Foad, 2009; Pollo et al., 2011), and cognitive performance (e.g. Parker et al., 2011; Weger and Loughnan, 2013). ‘Ergogenic placebos’ have been administered with the verbal suggestion that they can increase muscle power and endurance in weight lifters and cyclists (e.g. Beedie et al., 2006; Maganaris et al., 2000). Placebos introduced as ‘cognitive enhancers’ suggested improvement of attention or memory capacity during various learning tasks. In the above mentioned cases, the placebo was used to initiate a specific behavior and/or to increase the persistence in this activity. The placebo acted as a motivator to practice a certain behavior.

In the present investigation, we chose a placebo approach to increase adherence to practice a relaxation technique: progressive muscle relaxation (PMR, Jacobson, 1938). The participants were randomly assigned to one of two groups: a standard group (SG), which was instructed to practice PMR daily at home for two weeks, or a placebo group (PG), which practiced PMR for two weeks with additional daily placebo treatment. The placebo (sunflower oil) was introduced as a natural medicine to help the participants focus on their inner strengths and to mobilise their bodies’ natural relaxation powers.

To monitor adherence to relaxation practice, an app-assisted approach was used. The participants opened an app on their smartphones to listen to the 10-minute PMR instruction. Moreover, they were asked to rate their relaxation level directly before and after the PMR exercise. In this way, we were able to gather data on the frequency of PMR practice as well as on the experienced effectiveness of the relaxation exercise. It was hypothesised that the placebo would increase the quantity and quality of PMR practice.

## Method

### Participants

A total of 100 female University students with a mean age of 23.30 years (SD = 5.14) participated in this study. We included only women because of gender differences in placebo responsiveness (e.g. Ondo et al., 2013). Two women reported the diagnosis of a panic disorder, and one woman had a history of recurrent depression (currently in remission). Three participants reported using antidepressants. (Because exclusion of these participants did not change results, we report findings for the total sample). Of the participants, 79 reported no previous experience with relaxation techniques and none of them had practiced before regularly.

Written informed consent was obtained from all participants. The study was approved by the ethics committee of the university and carried out following the Declaration of Helsinki.

### Design

The participants were randomly assigned to one of two groups: a group with standard treatment (SG; *n* = 50), which participated in a two-week PMR course and a placebo group (PG; *n* = 50), which participated in the PMR course with additional daily placebo treatment. A random number table was used to allocate participants to the two treatment conditions (SG, PG). Each participant received an alphanumeric code which included the assigned condition (assuring allocation concealment). The two groups did not differ in mean age (SG = 23.44 years, SD = 5.89; PG = 23.16 years, SD = 4.32; *p* = 0.78), and reported psychological problems (as assessed by the Brief Symptom Inventory; Derogatis, 1993; *t*-score: SG: M = 54.48, SD = 12.60; PG: M = 55.02, SD = 11.25; *p* = 0.82).

The two groups did not differ in their attitude toward relaxation training (*‘Do you believe that relaxation techniques have positive/health-promoting effects?’* 0-6; 6 = strong belief; SG: M = 4.84, SD = 0.84; PG: M = 4.86, SD = 1.01; *p* = 0.91) and alternative medicine (‘*Do you believe that alternative/natural medicine has positive/health-promoting effects?*’ 0–6; 6 = strong belief; SG: M = 4.08, SD = 1.31; PG: M = 4.32, SD = 1.33; *p* = 0.36).

The placebo group received 30 ml sunflower oil provided in a blue glass bottle with a dropper (for oral administration). The oil was labeled ‘golden root oil’ (rhodiola rosea). Rhodiola rosea is a plant commonly found in regions with cold climates (e.g. Siberia) and has been used in traditional medicine for several applications (e.g. reduction of symptoms of stress, anxiety). For the present study, the oil was introduced as a natural medicine to help the participants focus on their inner strengths and to mobilise their bodies’ natural relaxation powers. Additionally, it was mentioned that the oil had already been tested in a clinical trial. The participants of the PG were instructed to take three drops of the placebo oil 10 minutes before the daily relaxation exercise (‘to allow absorption of the medicine’). None of the participants had already used rhodiola rosea before. At the end of the study, all participants were fully debriefed on the study design and use of the placebo.

### Procedure

The data acquisition for the study was conducted over 14 days. Before the course, the participants were instructed on how to practice PMR (abbreviated PMR training; Carlson and Hoyle, 1993). This was done in a first group session (with a maximum of 10 participants), which was conducted by a board-certified clinical psychologist with extensive experience in relaxation techniques and an assistant (master student). The women received an introduction to PMR and were taught how to tense and relax four different muscle groups (arms, shoulders/neck, face, legs). The guided PMR lasted about 10 minutes and was provided via a smartphone app (the same which was later used for practicing at home). The first group session took place separately for the PG and SG. In the first session, all participants completed a stress questionnaire (Kallus, 1995). The PG received the placebo oil and instructions on how to use it by the assistant.

The participants were instructed to carry out PMR daily at home. The adherence to the home practice of relaxation was monitored via the smartphone app. The handling of the app was explained in the first group session. The data gathering was achieved by combining a PWA (Progressive Web App) and a remote server for storage. The server was encrypted through an SSL connection. Participants first had to install the PWA onto their mobile phones and were then asked to answer three questions concerning their current emotional state (level of relaxation, pleasantness, arousal) using nine-point Likert scales (1–9; 9 = very pleasant, aroused, relaxed). After listening to the PMR audio file, the participants rated their emotional state again. The PMR instruction in the audio file was identical to the instruction in the group session. The survey was conducted via a webpage created with HTML, CSS, and Javascript (using the Vue.js Framework). The anonymous data were sent to a remote server where a Python Flask script handled the data collection and created a CSV file for each participant.

After the two-week relaxation course, a second group meeting took place (separately for the PG and SG). The participants were asked to rate the perceived effectiveness of PMR during the last two weeks (1–9, 9 = very effective) and to complete the stress questionnaire (Kallus,1995) again. The PG rated the perceived effectiveness of the placebo (‘*How effective was the golden root oil over the last two weeks?*’; 1–9; 9 = very effective). Additionally, the participants could comment on their experiences during home practice.

#### Questionnaire

The participants completed the Recovery-Stress Questionnaire (RSQ; Kallus, 1995) in the first and second group meeting. The questionnaire measures how individuals perceive their ability to deal with stressful situations. The questionnaire has seven stress-associated scales and five recovery-oriented scales with six items each (e.g. ‘I felt tired’, ‘I had a good time with friends’ (0–6; 0 = never, 6 = always).We computed the total stress score and the total recovery score for each participant. Possible mean scores range between 0 and 6 with higher scores indicating higher levels of stress and recovery. The Cronbach’s alpha of the total stress scale was .93/.96 and .91/.94 (first/second session) for the total recovery scale.

#### Statistical analysis

We conducted an intention-to-treat analysis where all participants who were randomly allocated to one group (SG, PG) were included in the statistical analysis and analysed according to the group they were originally assigned. According to the power analyzes via G*Power (3.1.9.7; Faul et al., 2007) a total sample size of 78 participants is sufficient to detect medium effects (Cohen’s *f* = .28) with a probability of 1–β = .8 (α = .05).

To test the effects of Group (SG, PG) on relaxation quantity (number of completed relaxation exercises) we computed a *t-*test.

Repeated measures analysis of variance (ANOVA) were performed to test the effect of Group (SG, PG) and Time (before/after relaxation exercise) on the reported average level of relaxation/arousal/pleasantness.

Additionally, ANOVAs were performed to test the effect of Session (before/after relaxation course) and Group (SG, PG) on the perceived level of stress and recovery (RSQ scores). We report η2p (partial eta^2^) as effect size measure. Significant effects were followed up with post-hoc *t-*tests.

Exploratory correlation analyses were conducted for the placebo group to test the association between the perceived effectiveness of the placebo and quantity/ quality of the relaxation exercises.

Finally, we performed a dropout analysis and compared participants who had finished vs. not finished the course (*t*-tests).

#### Data availability statement

The individual de-identified participant data that support the findings of this study are available on FigShare (statistical analysis with SPSS version 25).

## Results

**Figure 1.**
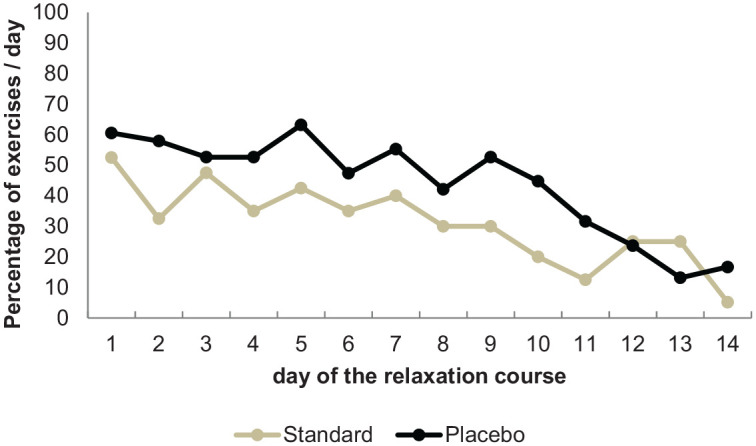
Percentage of completed relaxation exercises in the two groups. Footnote: 100%: *n* = 38 course completers in the placebo group; *n* = 40 course completers in the standard group.

*Relaxation quantity*: The PG (M = 6.24, SD = 2.79) had more completed relaxation exercises compared to the SG (M = 4.50, SD = 3.12; *t*(76) = 2.56, *p* < 0.013; Cohen’s *d* = 0.56). The daily compliance level (completed daily relaxation exercises over the two-week course) ranged between 63% and 13% in the PG, and between 53% and 5% in the SG. The compliance level decreased over time (see Figure 1).

*Relaxation quality*: For the arousal ratings the main effect Time (*F*(1,76) = 73.44, *p* < 0.001, η^2^*p* = 0.516) and the interaction Time × Group (*F*(1,76) = 5.25, *p* = 0.025, η^2^*p* = 0.071) were significant. The conducted post-hoc *t*-tests showed that the PG reported lower arousal than the SG before the relaxation exercise (*p* = 0.029), but not after the exercise (*p* = 0.25). In both groups, the arousal ratings decreased from the first to the second measurement (*p* < 0.001). The ratings are displayed in Table 1.

**Table 1.** Affective ratings (means, standard deviations) before and after the daily relaxation exercise in the two groups.

	**Placebo Group**		**Standard Group**	
	Before	After	Before	After
**Relaxation**	4.83 (1.26)	6.49 (0.92)	4.65 (0.99)	6.43 (0.78)
**Valence**	5.56 (1.09)	6.42 (0.92)	5.60 (1.05)	6.45 (1.04)
**Arousal**	2.67 (1.24)	2.09 (1.01)	3.36 (1.38)	2.37 (1.01)

For the average level of relaxation and pleasantness the main effect Time reached statistical significance. Participants reported a higher level of relaxation and pleasantness after the PMR exercise than before (relaxation: *F*(1,69) = 193.34, *p* < 0.001, η^2^*p* = 0.737; pleasantness: *F*(1,69) = 140.19, *p* < 0.001, η^2^*p* = 0.670). The main effects group and the interactions were not significant (*p* > 0.54).

The two groups (PG, SG) did not differ in the perceived effectiveness of PMR after the two-week course (*p* = 0.52; M_total_ = 5.36, SD = 1.87).

*Reported stress/ recovery level*: The ANOVA for the total stress score did not reveal significant results (*p* ⩾ 0.069). For recovery, the main effect Session reached statistical significance (*F*(1,76) = 5.65, *p* = 0.02, η^2^*p* = 0.069). Participants reported a higher recovery level after the two-week relaxation course than before (see Table 2). The main effect group and the interaction were not significant (*p* > 0.24).

**Table 2.** Perceived stress and recovery level (means, standard deviations) before and after the relaxation course in the two groups.

	**Placebo group**		**Standard group**	
	Before	After	Before	After
**RSQ_Stress**	2.05 (.70)	1.83 (.86)	1.95 (.87)	1.81 (.86)
**RSQ_Recovery**	2.69 (.70)	3.01 (.84)	2.96 (.74)	3.09 (.79)

Footnote: RSQ: Recovery-Stress-Questionnaire.

*Drop out analysis*: Twelve participants of the placebo group and 10 participants of the standard group did not show up to the second group meeting at the end of the two-week relaxation course. These participants were therefore excluded from the analysis.

Course completers (*n* = 78) and dropouts (*n* = 22) differed from each other. Those who did not show up to the second group meeting reported more psychological problems (BSI *t*-score: M = 59.27, SD = 11.23) and a higher stress level in the first group session (RSQ-total stress: M = 2.65, SD = 0.95) than those who completed the course (BSI *t*-score: M = 53.47, SD = 11.23; *t*(98) = 2.05, *p* = 0.043; RSQ-total stress: M = 2.00, SD = 0.80; *t*(98) = 3.24, *p* = 0.002). The two groups did not differ in their general recovery level (RSQ- total recovery) in the first session (*p* = 0.09).

*Exploratory correlation analyses for the placebo group*: The rated effectiveness of the placebo (M = 3.58, SD = 1.81, range: 1–8) showed a marginally significant correlation with ‚relaxation quantity’ (*r* = 0.27, *p* = 0.098). Moreover, a higher rating for placebo effectiveness was associated with a greater relaxation increase after the PMR exercise (difference of relaxation level after minus before the exercise). The correlation was *r* = 0.36 (*p* = 0.028). The perceived placebo effectiveness was positively correlated with the perceived effectiveness of PMR (*r* = 0.69, *p* < 0.001).

## Discussion

This study showed that a placebo can help to improve adherence to relaxation instructions. The placebo group that received ‘a natural medicine for the activation of the body’s natural relaxation response’ practiced PMR more often than a standard group without a placebo. In contrast, the placebo did not influence the quality of the relaxation response. The two groups (placebo, standard) did not differ in the reported average relaxation level after a training unit. However, the increase in relaxation level due to the PMR exercise was positively associated with the perceived effectiveness of the placebo. These findings are in line with previous placebo research demonstrating that the evaluation of the placebo is connected with the placebo effect. When the placebo is perceived as a real treatment, this facilitates placebo responsivity and increases the placebo effect (e.g. Zorjan et al., 2019).

The findings of the present investigation raise basic questions regarding the opportunities and limits of placebo treatments. It is known that placebos show differential effectiveness depending on the particular symptom and sample being treated. For example, substantial placebo effects have been found in the treatment of some disorders (e.g. depression, irritable bowel syndrome) but not in others (e.g. the common cold) (Hróbjartsson and Gøtzsche, 2001). Substantial placebo effects have also been observed in healthy individuals. For example, Schienle et al. (2014) administered a placebo labeled as an anti-nausea drug to participants, while they were presented with stimuli commonly perceived as repulsive (e.g. images of spoiled food, excrements). The placebo reduced the intensity of disgust by more than half of its original value. The mentioned examples show that marked distress about having the symptoms and the need for change are preconditions for a placebo to be effective. Depressed patients and disgusted individuals want the negative affective state to end. They have a desire for symptom improvement which is positively correlated with the magnitude of the placebo effect (e.g. Enck et al., 2008). On the other hand, healthy students with a moderate stress level (as in the present investigation) do not need a change in the same degree.

Moreover, it has been shown that placebos can even negatively affect the motivation and effort to show a specific behavior. For example, Tippens et al. (2014) found that participants with stronger beliefs in a placebo (100% expectation to have received a dietary supplement for weight loss) reported a decline in self-efficacy throughout the study and tended to lose a smaller proportion of weight than participants with less pronounced beliefs. Similar findings have been reported by Höfler et al. (2019). The participants received sham transcranial magnetic stimulation that was administered along with the verbal suggestion that the treatment would increase visual attention. In this eye-tracking study with healthy participants, the placebo did not affect the performance level. The placebo reduced the effort to complete the visual search task because the participant expected that the placebo would do the ‘work’ for them. These findings point to the high relevance of verbal placebo suggestions to stimulate self-efficacy and motivation in treated individuals.

It seems promising to test the ‘relaxation placebo’ in clinical groups that typically receive relaxation training as one component of psychotherapy (e.g. patients with depression, anxiety disorders). Here, greater placebo effects can be expected because of the greater need for positive change. Additionally, this type of treatment is easy to administer and cost-effective. On the other hand, associated risks and disadvantages of placebo treatment in the context of psychotherapy should not be neglected. Honesty and transparency are two key components of the ethical framework for the counseling professions. Both characterize a positive patient-therapist relationship, which makes a substantial and consistent contribution to the (long-term) psychotherapy outcome. Therefore, the debriefing procedure (after the termination of placebo treatment) appears to be extremely important, to prevent a reduction of the patient's experience of therapy success and trust in the therapist. As an alternative, an open-label placebo strategy could be used that has already been successfully applied in various areas (for a review see Colloca and Howick, 2018).

The findings of this study have to be seen in light of some limitations. The placebo administration preceded the relaxation exercise by 10 minutes. The instruction to wait with the relaxation exercise had been given to enhance the credibility of the cover story; the delay should have helped the ‘golden root oil’ to take effect (‘drug absorption’). However, the placebo had an unintended immediate effect. (This was also mentioned in the last group session by some of the participants of the PG, who noted the instant effect of the ‘golden root oil’). As a consequence, the placebo group reported less arousal compared to the standard group already before the relaxation exercise. For future investigations, it would be important to include a third measurement point in the design to capture arousal before administering the placebo.

Moreover, the study had a relatively high dropout rate (22%), which did not differ between treatment arms (PG, SG). However, participants who dropped out reported more psychological problems and a higher stress level. This group would have been the target group for PMR. The placebo could not prevent dropout.
